# Effects of psychophysiological stress on perceptual responses during low-volume high intensity interval exercise: insights from ACTH and cortisol in overweight-to-obese adults

**DOI:** 10.3389/fphys.2026.1762153

**Published:** 2026-03-13

**Authors:** Ruohan Zhang, Jintao Guo, Jingyuan Sun, Jinfa Gu

**Affiliations:** 1 Exercise and Sports Science Programme, School of Health Sciences, Universiti Sains Malaysia, Kota Bharu, Kelantan, Malaysia; 2 School of Competitive Sports, Beijing Sport University, Beijing, China

**Keywords:** cardiorespiratory fitness, low-volume high intensity interval exercise, overweight and obesity, perceptual responses, psychophysiological stress

## Abstract

**Background:**

Low-volume high-intensity interval exercise (Lv-HIIE) is a time-efficient training strategy, but little is known about how psychophysiological stress, as reflected by endocrine markers, is associated with perceived exercise experiences in overweight-to-obese adults. More specifically, research on ACTH- and cortisol-related stress responses during multi-week Lv-HIIE is very limited, and no existing studies have examined how these hormonal patterns are related to perceptual outcomes.

**Purpose:**

This study examined the associations of ACTH and cortisol responses with perceptual outcomes during a 10-week Lv-HIIE intervention and whether these associations changed across repeated sessions.

**Methods:**

Thirty-two inactive adults (11 males, 21 females; 28.3 ± 4.9 years) with overweight or obesity completed 30 Lv-HIIE sessions over 10 weeks, HIIE consisted of 8 × 1-min work intervals performed at 90% of maximal aerobic speed (MAS), separated with 75-s recovery periods. Perceptual responses (affective valence, arousal, perceived exertion, perceived recovery, and enjoyment) and stress markers (ACTH, cortisol) were collected at sessions 1, 15, and 30. Heart-rate responses and body-composition measures were also assessed.

**Results:**

Lv-HIIE produced small but significant improvements in body composition and aerobic fitness (%BF, WHR, V̇O_2_max, MAS; all p < 0.01). Physiological strain decreased, with HR and %HRmax lower in S15 and S30 than S1 across most work intervals (p < 0.05; ES = 0.39–1.73). Affective valence improved from negative in S1 (−0.53 ± 0.44) to positive in S15 and S30 (p < 0.001; ES = 0.48–1.29), while RPE decreased and perceived recovery increased (p < 0.02; ES = 0.47–2.86). Enjoyment also increased (PACES: 97.0 → 110.8; p < 0.001; ES > 0.67). ACTH and cortisol showed positive correlations with HR and RPE (r = 0.40–0.61; p < 0.03) and negative correlations with affective valence, recovery, and enjoyment (r = −0.36 to −0.56; p < 0.05).

**Conclusion:**

Endocrine stress markers (ACTH and cortisol) were significantly associated with perceptual responses during the 10-week Lv-HIIE, with the strength of their correlations changing across the exercise period. These findings suggest that Lv-HIIE may promote improved perceptual tolerance and affective adaptation during training in adults with excess body weight.

## 1 Introduction

Obesity is a major global problem. The World Obesity Report (2025) indicates that the number of adults with obesity is expected to rise from 524 million in 2010 to 1.13 billion by 2030, with nearly 50% of the global adult population affected by overweight and obesity (World Obesity Federation, 2025). Moreover, The Lancet projects that the age-standardized prevalence of overweight and obesity will increase by 30.7% over the next 30 years, and by 2050 nearly 60% of adults will be overweight or obese (Ng et al., 2025). Against this backdrop, the World Health Organization (2024) reports that 31% of adults fail to meet recommended physical activity levels, with time constraints cited as the most significant barrier (Hoare et al., 2017; Heesch and Mâsse, 2004; Cavallini, 2022; Atakan et al., 2021). For adults with excess body weight or obesity aiming to reduce body fat, traditional steady-state moderate-intensity activities are associated with minimal weight loss (Shaw et al., 2006; Wu et al., 2009). These limitations have stimulated interest in more efficient exercise approaches, with high-intensity interval exercise (HIIE) being considered a promising option (Gillen and Gibala, 2014). Although HIIE confers well-documented health benefits, including improvements in cardiovascular health and reductions in body weight (D’Amuri et al., 2021; Wewege et al., 2017; Turk et al., 2017), adults with obesity may experience emotional resistance or psychological stress toward HIIE, highlighting the need to consider exercise-related perceptual responses such as affective valence, perceived exertion and enjoyment.

In this context, perceptual responses during exercise have been widely studied for their motivational significance (Martinez et al., 2015; Ekkekakis et al., 2008; Ekkekakis et al., 2011). These responses are particularly important in the context of HIIE, which presents unique challenges due to its rigorous nature and may cause significant discomfort in overweight-to-obese adults because of greater physical demands compared to moderate-intensity continuous exercise (MICE) (Weston et al., 2014; Hardcastle et al., 2014; Niven et al., 2021). While such discomfort is often attributed to cardiorespiratory strain, perceived exertion can also profoundly affect feelings of pleasure or displeasure during activity, and personal goals as well as the surrounding exercise environment shape affective responses, which cannot be fully captured by cardiorespiratory metrics alone. Thus, careful consideration is required to avoid negative affective experiences that may impair long-term adherence, especially in low-fitness populations (Follador et al., 2018).

HIIE has gained popularity as a time-efficient strategy to improve cardiorespiratory fitness, body composition, and metabolic health in overweight and obese adults, while also reducing risk indicators for cardiovascular and metabolic diseases (Andreato et al., 2019; Batacan et al., 2017; Wewege et al., 2017). However, high-volume HIIE cycles have been proven to lead to lower pleasure and enjoyment compared to MICE, particularly in low-active women with obesity (Decker and Ekkekakis, 2017). Although low-volume HIIE (Lv-HIIE) may offer similar benefits, its effects on perceptual responses in overweight-to-obese adults remain unclear. Prior studies have mainly focused on observing cardiorespiratory responses (e.g., heart rate, oxygen consumption) to explain exercise-induced affect. Nevertheless, this focus may be insufficient to explain perceptual outcomes. These measures may overlook critical psychophysiological factors that also influence emotional experience, exertion, recovery, and enjoyment. Moreover, stress-related responses such as muscle discomfort and fatigue are not adequately captured by cardiorespiratory measures alone. In contrast, psychophysiological stress responses may offer insights into mechanisms of exhaustion that diminish positive affect. Supporting this view, previous investigation in adolescents suggested that physiological factors can shape exercise-induced feelings during HIIE (Malik et al., 2021). Nevertheless, whether changes in perceptual responses (pleasure, arousal, enjoyment, perceived exertion, and recovery) can be explained by psychophysiological stress in overweight-to-obese adults remains unknown.

Given these challenges, researchers have explored improved forms of HIIE to retain benefits while reducing intensity and physiological demands. Lv-HIIE has emerged as a promising alternative to traditional HIIE, offering comparable health improvements with shorter, less intense regimens (Sabag et al., 2022; Yin et al., 2024). This approach may reduce discomfort and enhance sustainability among adults living with obesity. Moreover, regulating psychophysiological stress may be crucial for supporting more positive perceptual experiences (higher affective valence and enjoyment, lower perceived exertion) and ultimately improving adherence.

However, no study has systematically examined how psychophysiological stress markers (ACTH and cortisol) interact with perceptual responses across the full course of a multi-week HIIE intervention in adults with excess body weight. In the present study, psychophysiological stress was operationalized primarily through endocrine stress markers (ACTH and cortisol), which reflect systemic stress responses and are conceptually distinct from cardiorespiratory strain and local muscular fatigue. Furthermore, it remains unclear whether these stress–perception relationships shift as individuals progress from initial exposure to mid-program strain and eventual adaptation. Addressing this gap, this study is the first to track stress–perception coupling at three key time points (sessions 1, 15, and 30) during a 10-week Lv-HIIE programme.

Therefore, this study aims to investigate the effects of psychophysiological stress on various perceptual responses during Lv-HIIE in overweight-to-obese adults. Specifically, it focuses on perceptual responses (pleasure, arousal, enjoyment, perceived exertion, and recovery) alongside cardiorespiratory responses (heart rate, oxygen consumption), with the goal of determining whether psychophysiological stress (e.g., cortisol-related responses) influences these outcomes and informs the feasibility and sustainability of Lv-HIIE as a public health strategy for adults with obesity and excess body weight.

## 2 Materials and methods

### 2.1 Participants

Thirty-two adults (N = 32) volunteered to participate in this study. The required sample size was estimated *a priori* using G*Power (version 3.1.9.7) based on a repeated-measures ANOVA framework, assuming a medium effect size (f = 0.30), an alpha level of 0.05, and statistical power of 0.80. This effect size corresponded to the within-subject main effect of time across sessions, rather than to any specific perceptual outcome or endocrine marker (e.g., ACTH or cortisol). The calculation indicated that a minimum of 31 participants would be required; therefore, the final sample of 32 met and slightly exceeded this requirement.

The study was conducted at the Sports Complex, Health Campus, Universiti Sains Malaysia. Participants were recruited from students and staff via campus email announcements and posters. Eligibility was screened prior to enrolment, all participants provided written informed consent. Inclusion criteria were male and female adults aged 20–35 years; a body mass index (BMI) between 23.0 and 30.0 kg m^-2^; medically cleared to engage in exercise; and physically inactive, defined as not meeting the recommended ≥150 min week^-1^ of moderate-intensity physical activity. BMI categories were defined according to the Asia-Pacific classification, with BMI 23.0–24.9 kg m^-2^ classified as overweight and BMI 25.0–30.0 kg m^-2^ classified as obesity. Exclusion criteria included smoking; metabolic conditions (e.g., hypertension, dyslipidaemia, or hyperglycaemia); use of medications or substances affecting cardiorespiratory or metabolic responses; recent participation in structured training (within 6 months); and exercise contraindications identified by the Physical Activity Readiness Questionnaire (PAR-Q). Ethical approval for the study was obtained from the Human Research Ethics Committee of Universiti Sains Malaysia (JEPeM Code: USM/JEPeM/22080549).

The sample included a higher proportion of female participants (21 females, 11 males). Sex-specific factors that may influence endocrine responses, including menstrual cycle phase and related hormonal variability, were not monitored or controlled as part of the study design.

### 2.2 Experimental overview

**FIGURE 1:**
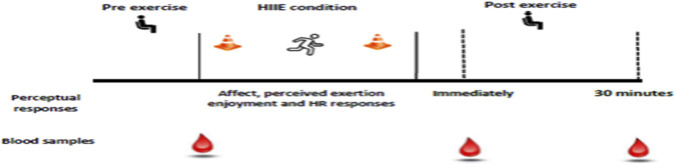
Summary of experimental design.

This interventional study employed a single-arm, within-subjects design to examine within-participant changes over time and to explore associations between psychophysiological stress markers and perceptual responses during repeated exposure to a low-volume high-intensity interval exercise (Lv-HIIE) program. Participants completed a 10-week Lv-HIIE intervention consisting of three sessions per week (30 sessions in total), with at least 48 h of rest between sessions. Before the training intervention, participants underwent baseline assessments including anthropometric measurements, cardiorespiratory fitness, and a 20-m shuttle run test (pre-HIIE). Following the intervention, participants repeated the shuttle run test (post-HIIE) to evaluate training effects. Perceptual responses (affective valence, felt arousal, and rating of perceived exertion) and heart rate were recorded during each Lv-HIIE session, while perceived enjoyment was assessed after the first, 15th, and 30th sessions. Heart rate recovery and perceived recovery status were also measured after each session. Blood samples for ACTH and cortisol were collected before, immediately after, and 30 min post-exercise at the first, 15th, and final sessions (illustrated in Figure 1). All exercise sessions were performed at the Sports Complex, Health Campus, Universiti Sains Malaysia, at the same time of day (08:00–12:00) to minimize environmental and circadian influences. Participants were instructed to abstain from other physical activity during the training program.

### 2.3 Anthropometric and physical activity measures

Body mass and stature were measured to the nearest 0.1 kg and 0.1 cm, respectively (the participants were shoeless and wore light clothing) by using (Seca 220, Hamburg, Germany). Waist-to-hip ratio (WHR) was also determined by dividing waist (cm) by hip circumference (cm). Body mass index (BMI) was calculated as body mass (kg) divided by stature (m) squared. A “foot-to-foot” body composition analyzer (Tanita model TBF-140, Japan) was used to measure percentage of body fat (%BF). Percentage body fat (%BF) was determined using bioelectrical impedance analysis (BIA) as evidence has suggested that BIA provides a relatively accurate prediction of %BF in overweight/obese adults (Li et al., 2022).

Participants’ daily habitual physical activity was measured by using the English language version of the International Physical Activity Questionnaire (IPAQ-M; Shamsuddin et al., 2015), which comprised of 12 items, covering vigorous, moderate, walking, sitting, and sleeping activities. The validity and reliability of the IPAQ-M had been established previously in the Malaysian adult population (Chu and Moy, 2015). Each participant also completed a physical activity readiness questionnaire (PAR-Q) and a physical activity before initiating the study. IPAQ-M can be classified into three levels of categorical score that consists of Category 1 (Inactive; <600 MET-min/week), Category 2 (moderately active; <3000 MET-minutes/week) and Category 3 (health-enhancing physical activity (HEPA); >3000 MET-minutes/week) (Macfarlane et al., 2007).

### 2.4 Cardiorespiratory fitness

Participants were familiarized with the treadmill before completing an incremental speed-based protocol to determine maximal oxygen uptake (*V̇*O_2_max) and ventilatory threshold (VT). The test began with a 3 min warm-up at 4.0 km/h, followed by increments of 0.5 km/h every 30 s starting at 5.0 km/h until volitional exhaustion, and ended with a 5 min cool down. The treadmill gradient was fixed at 1% to reflect outdoor running energy cost (Jones and Doust, 1996). *V̇*O_2_max was determined using standard criteria, including a plateau in oxygen uptake, respiratory exchange ratio ≥1.10, and HRmax within five beats of age-predicted HRpeak. This incremental protocol has previously been validated in overweight/obese adults (Balcı, 2012; Khammassi et al., 2018). Gas exchange and heart rate responses were recorded as they served as compliance criteria for the Lv-HIIE protocol (≥90% HRmax).

### 2.5 HIIE protocols

Participants completed a 10-week Lv-HIIE training intervention consisting of a 3-min warm-up at 4.0 km h^-1^, followed by 6–10 repetitions of 1-min running intervals at 90% of MAS, as determined from the 20-m shuttle run test (SRT) (Table 1).

**TABLE 1:** 10-week exercise program for Lv-HIIE.

Training groups	Variables	Week 1–2	Week 3–4	Week 5–6	Week 7–8	Week 9–10
Lv-HIIE	Repetition	6	7	8	9	10
	Duration work/recovery	60/75s				
	Work intensity	90% of MAS				
	Recovery intensity	Self-paced				

The relative intensity was maintained at 90% of MAS throughout the intervention to standardize the external workload across sessions. This design allowed changes in internal load (e.g., heart rate, perceived exertion, and endocrine stress responses) to reflect physiological and perceptual adaptation over time, rather than adjustments in prescribed exercise intensity resulting from fitness improvements.

**FIGURE 2:**
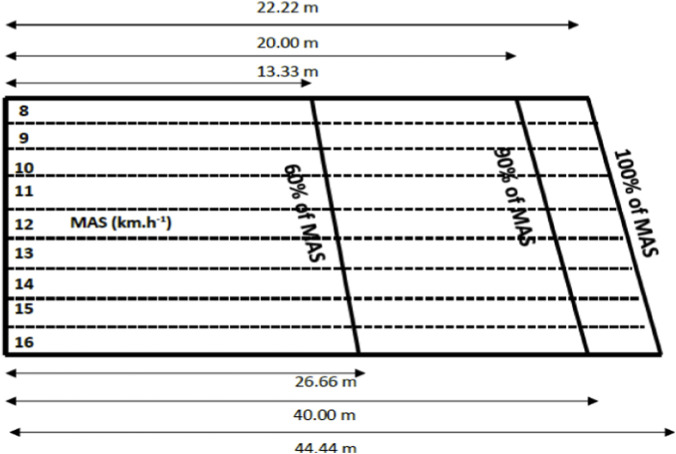
Short track for interval exercise.

Each work interval was interspersed with 75 s of active recovery at self-paced walking. To ensure correct pacing, cones were positioned according to each participant’s individual running distance, with a whistle blow emitted every 10 s to guide participants to the cone (Figure 2). A 2-min cool down at self-paced running was provided at the end of the protocol. Participants performed Lv-HIIE three times per week for 10 weeks (total of 30 sessions). Compliance with the HIIE protocol was verified using a ≥85% HRmax cutoff point as the criterion for satisfactory adherence.

### 2.6 Experimental measures

Prior to the experimental sessions, participants underwent a standardized familiarization procedure for all perceptual assessment scales used in the study, including the FS, FAS, RPE, and PRS. During this procedure, standardized verbal explanations of each scale and their numerical anchors were provided, and participants completed practice ratings during a brief low-intensity exercise bout to ensure comprehension before data collection.

#### 2.6.1 Gas exchange and heart rate

Pulmonary gas exchange and HR were measured continuously during the incremental test using a calibrated metabolic cart (Cortex Metalyzer III B, Leipzig, Germany) and a telemetry system (Polar Electro, Kempele, Finland), respectively. Both gas exchange and HR data were averaged over 10 s intervals. The VT was estimated at the point where the first disproportionate increase in CO_2_ production compared to VO_2_ occurred and was verified using the ventilatory equivalents for carbon dioxide production (VCO_2_) and VO_2_. VO_2max_ and maximal HR (HRmax) were defined as the highest 10-s average in VO_2_ and HR elicited during the incremental test to exhaustion. HR responses including both working heart rate and recovery heart rate were measured continuously at each session across a 10-week Lv-HIIE intervention.

#### 2.6.2 Affective responses

Affective valence (pleasure/displeasure) was measured using the feeling scale (FS; Hardy and Rejeski, 1989) according to previous work in overweight/obese adults (Ekkekakis, 2009). Participants responded to how they felt on an 11-point bipolar scale ranging from “Very Good” (+5) to “Very Bad” (−5). In the present study, the FS demonstrated acceptable internal consistency across administrations (Cronbach’s α = 0.89). Perceived activation levels were measured using the single item felt arousal scale (FAS; Svebak and Murgatroyd, 1985). Participants were asked to rate themselves on a six-point scale ranging from 1 ‘low arousal’ to 6 ‘high arousal’. In the present study, the FAS demonstrated acceptable internal consistency across administrations (Cronbach’s α = 0.87). FS and FAS exhibited correlations ranging from 0.41 to 0.59 and 0.47 to 0.65, respectively, with the Affect Grid (Russell et al., 1989), indicative of convergent validity with similar established measures (Landuyt et al., 2000). Participants responded to the FS and FAS 5 min before exercise; 20 s before the end of the warm-up session; and 20 s before the end of each work.

#### 2.6.3 Rating of perceived exertion

The 10-point Category-Ratio 10 Scale (CR-10; Borg, 1998), also commonly referred to as the Rating of Perceived Exertion (RPE), was used to assess participants perceived effort during exercise. The CR-10 is a 10-point scale ranging from 0 to 10, with anchors ranging from “No exertion at all” (0) to “Maximal exertion” (10). The RPE data were collected at the same during-interval time points as the FS. Participants respond to the questions, “How hard your effort during exercise” via a 010-point Likert item ranging from 0 (rest) to 10 (maximal).

#### 2.6.4 Post-exercise enjoyment

Participants’ enjoyment of each exercise condition was examined using the Physical Activity Enjoyment Scale (PACES; Kendzierski and DeCarlo, 1991) 10 min post-exercise. This 18-item measure was scored on a seven-point bipolar scale. Rate how you feel about the physical activity (i.e., HIIE protocol) you have been doing” based on a scale of one through 7. The score for 18 items was summed to calculate a total enjoyment score out of 126 for each exercise condition. The internal consistency was acceptable at each administration (Cronbach’s αs > 0.90) in the present study. PACES were recorded following 10 min of each exercise session on the first, 15th, and final days of the Lv-HIIE intervention.

#### 2.6.5 Perceived recovery status

The Perceived Recovery Scale (PRS) was a single-item, 0- to 10-point scale with two sets of verbal anchors defining the numerical indicators of both recovery and expected performance, where 0 = very poorly recovered (poor performance) and 10 = fully recovered (optimal performance). This scale had a clear construct of assessment (recovery) and was developed in a sample representing the target population. Questions remained over the application of the PRS in practice, and thus any further scrutiny of this scale was considered useful to those implementing subjective measures to quantify training effects.

#### 2.6.6 Blood processing

Blood samples were obtained by venipuncture into vacuum tubes with a gel separator (5 mL; BD Vacutainer, Franklin Lakes, NJ, United States). The samples were allowed to clot, and the serum was separated by centrifugation at room temperature. The serum samples were aliquoted and stored at −70 °C until analysis. The concentrations of human cortisol and ACTH were measured in serum. The concentrations of these hormones were analyzed using commercial enzyme-linked immunosorbent assay (ELISA) kits. All blood samples were collected on the first, 15th, and final days of the Lv-HIIE intervention before exercise, immediately after exercise, and 30 min post-exercise, and ACTH and cortisol concentrations used for correlation analyses were calculated as the mean of these three time points at each session to represent overall psychophysiological stress across the exercise bout.

### 2.7 Data analysis

All the statistical analysis was conducted via SPSS (SPSS 28.0; IBM Corporation, Armonk, NY, USA). Descriptive characteristics (means ± standard deviations) between the pre-test and post-test were analyzed via paired samples t tests. The Shapiro‒Wilks test was used to test the normality of distribution for the dependent variables. One-way repeated-measures ANOVA was used to examine differences in variables measured during the pre-exercise, warm-up, and cool-down periods, as well as post-exercise enjoyment, across the three selected Lv-HIIE sessions (S1, S15, and S30). For variables measured during the exercise phase, a two-way repeated-measures ANOVA (session × work interval) was used to examine differences in affective responses, heart rate, ratings of perceived exertion, perceived recovery status, and ACTH and cortisol levels across the three Lv-HIIE sessions. To satisfy the assumptions of the repeated-measures design, analyses were restricted to equivalent work intervals common to all sessions. In the event of significant effects, follow-up pairwise comparisons were conducted to examine the location of the mean differences. The magnitude of the mean differences was interpreted via the effect size (ES) calculated via Cohen’s d (Cohen, 1988), where an ES of 0.20 was considered a small change between means, and 0.50 and 0.80 were interpreted as moderate and large changes, respectively. Pearson’s product‒moment correlation coefficients (r), which range from −1 to +1, were calculated to assess the correlations between perceptual responses and the hormone levels of ACTH and cortisol during Lv-HIIE. For correlation analyses, ACTH and cortisol concentrations were calculated as the mean of the pre-exercise, immediate post-exercise, and 30-min post-exercise values at each session. A coefficient near +1 indicates a strong positive correlation, a coefficient near −1 indicates a strong negative correlation, and a coefficient near 0 indicates no correlation. All analyses were conducted to examine within-subject changes over time and associations between variables; no causal inferences were intended or drawn from the present study design.

## 3 Results

A total of 40 overweight-to-obese adults were initially recruited for the study. Eight participants withdrew during the intervention due to injury (n = 3), personal reasons (n = 2), or scheduling conflicts related to academic or work commitments (n = 3). Consequently, 32 participants completed all phases of the study and were included in the final analyses. All participants included in the analyses completed all 30 scheduled Lv-HIIE sessions, resulting in a session attendance rate of 100% among completers. Compliance with the prescribed exercise intensity was high, with participants meeting the target criterion (≥85% HRmax) in the majority of training sessions.

Participants’ physical activity levels, assessed using the International Physical Activity Questionnaire (IPAQ), indicated that the sample was generally low-active (mean = 518 ± 334 MET·min/week). According to the WHO Asian BMI classification, 15.6% of participants were categorized as overweight (BMI = 23–24.9 kg·m^2^), while 84.4% were classified as living with obesity (BMI ≥25 kg·m^2^). Anthropometric and physiological measurements were collected at baseline, mid-intervention, and post-intervention to characterise participants’ physical profiles across the 10-week programme. These descriptive indicators were used to contextualise the psychophysiological and perceptual responses examined in this study.

Anthropometric and physiological characteristics measured at baseline, mid-intervention, and post-intervention are presented in Table 2. Descriptive values are provided for body weight, BMI, body-fat percentage, waist–hip ratio, maximal aerobic speed (MAS), estimated *V̇*O_2_max, and HRmax.

**TABLE 2:** Descriptive characteristics of the participants (N = 32).

Variables	Pre-test (Mean ± SD)	Mid-test (Mean ± SD)	Post-test (Mean ± SD)	p-value	F-value/ES (Cohen’s d)
Body weight	74.19 ± 11.22	73.92 ± 10.78	73.93 ± 11.25	0.63	0.45
BMI	28.21 ± 2.93	28.08 ± 2.8	28.12 ± 2.85	0.61	0.45
WHR	0.81 ± 0.08	0.79 ± 0.09	0.78 ± 0.073*	0.013	5.22
%BF (%)	32.89 ± 5.39	32.04 ± 5.31	31.78 ± 5.51*	0.003	6.98
MAS	9.55 ± 0.68	-	10.03 ± 0.76^	<0.001	1.80 [1.23, 2.36]
$\overset{˙}{V}$ O_2_max	38.15 ± 2.28	-	38.55 ± 2.44^	0.002	0.62 [0.24, 0.99]
HR_max_	184 ± 10.25	-	180.63 ± 9.31^	<0.03	0.43 [0.06, 0.79]

Values are reported as mean ± standard deviation (SD). F-values are from repeated-measures ANOVA. Effect sizes (ES) for Pre–Post comparisons are reported as Cohen’s d with 95% confidence intervals (CIs). Abbreviations: BMI, body mass index; BF, body fat; WHR, waist hip ratio; $\overset{˙}{V}$ O_2_max, Maximal oxygen uptake; MAS, maximum aerobic speed; HRmax, maximum heart rate; *Significant differences between all-time points (p < 0.05); ^Significant difference between pre- and post-intervention (p < 0.05).

### 3.1 Heart rate responses

**FIGURE 3:**
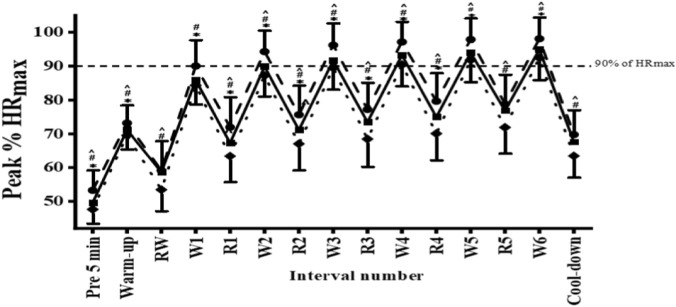
% HR_max_ S1 (s1; ●), S15 (s15; ■) and S30 (s30; ♦). Abbreviations: W = work interval and R = recovery interval. *Significant difference between S1 and S15 (p < 0.05), #Significant difference between S1 and S30 (p < 0.05), ^Significant difference between S15 and S30 (p < 0.05). Error bars are presented as standard deviation.

HR percentage during the three sessions of Lv-HIIE conditions are illustrated in Figure 3. Changes in %HR were examined separately for different periods. During the pre-5 min, warm-up, and cool-down periods, one-way repeated-measures ANOVAs revealed significant session effects (pre-5 min: F (2,62) = 14.92, p = 0.000; warm-up: F (2,62) = 8.24, p = 0.001; cool-down: F (2,62) = 14.16, p = 0.000), indicating lower %HR values with training progression.

During the exercise period, %HR during work intervals was analyzed using a two-way repeated-measures ANOVA with Session (S1, S15, S30) and Interval (w1–w6) as within-subject factors. The analysis revealed a significant main effect of Session (F (2,62) = 42.61, p = 0.000) and a significant main effect of Interval (F (5,155) = 215.79, p = 0.000), whereas the Session × Interval interaction was not significant (F (10,310) = 1.28, p = 0.242). Post hoc comparisons indicated that %HR during work intervals was consistently higher in S1 than in S15 across intervals w1–w6 (all p ≤ 0.001, ES = 0.66–1.00) and higher than in S30 across all work intervals (all p < 0.001, ES = 0.89–1.79). In addition, %HR in S15 was higher than in S30 at work intervals w2–w6 (all p < 0.01, ES = 0.54–0.73).

During the recovery intervals, %HR was further analyzed using a two-way repeated-measures ANOVA with Session (S1, S15, S30) and Interval (rw, r1–r5) as within-subject factors. The analysis revealed significant main effects of Session (F (2,62) = 35.39, p = 0.000) and Interval (F (5,155) = 286.58, p = 0.000), as well as a significant Session × Interval interaction (F (10,310) = 2.82, p = 0.002). Post hoc analyses showed that %HR during recovery was higher in S1 than in S15 at intervals r1–r4 (all p < 0.01, ES = 0.59–0.76), whereas no significant differences were observed at rw or r5. In contrast, %HR during S1 was consistently higher than during S30 across all recovery intervals (all p < 0.01, ES = 0.64–1.58). Additionally, %HR during S15 was higher than during S30 across all recovery intervals (all p < 0.01, ES = 0.61–0.95), indicating enhanced cardiovascular recovery with exercise progression.

### 3.2 Affective responses

**FIGURE 4:**
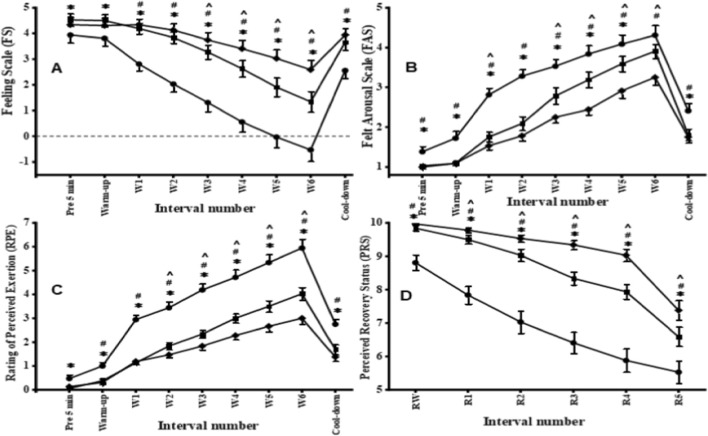
FS **(A)**, FAS **(B)**, RPE **(C)**, PRS **(D)**. S1 (s1; ●), S15 (s15; ■) and S30 (s30; ♦). Abbreviations: W = work interval; R = recovery interval. *Significant difference between S1 and S15 (p < 0.05), #Significant difference between S1 and S30 (p < 0.05), ^Significant difference between S15 and S30 (p < 0.05). Error bars are presented as standard deviation.

Affective valence (FS) responses during the three Lv-HIIE sessions are shown in Figure 4A. Changes in FS were examined separately for different periods. During the pre-5 min, warm-up, and cool-down periods, one-way repeated-measures ANOVAs revealed significant session effects (pre-5 min: F (2,62) = 15.33, p = 0.000; warm-up: F (2,62) = 8.34, p = 0.001; cool-down: F (2,62) = 14.08, p = 0.000). Post hoc analyses indicated that FS values during S30 were significantly higher than those during S1 across all three periods (all p < 0.01), whereas no significant differences were observed between S1 and S15 or between S15 and S30 (all p > 0.05). During the exercise period, FS was further analyzed using a two-way repeated-measures ANOVA with Session (S1, S15, S30) and Interval (1–6) as within-subject factors. A significant Session × Interval interaction was observed (F (4.06, 125.99) = 11.56, p < 0.001). Post hoc comparisons revealed that FS was lower in S1 than in S15 across all work intervals (p < 0.02, ES = 0.48–1.05) and lower than in S30 at work intervals 1–6 (p < 0.001, ES = 1.15–1.29). In addition, FS in S15 was lower than in S30 at work intervals 3–6 (p < 0.01, ES = 0.49–0.85). Across sessions, FS declined significantly across work intervals (all p < 0.001); however, the onset of this decline occurred progressively later with training (S1: from interval 1; S15: from interval 2; S30: from interval 4). At the final work interval, FS remained positive in all participants during S30 (1.16 ± 0.46), in most participants during S15 (0.41 ± 0.43), but was negative in all participants during S1 (−0.53 ± 0.44).

FAS responses during the three Lv-HIIE sessions are presented in Figure 4B. Changes in FAS were examined separately for different periods. During the pre-5 min, warm-up, and cool-down periods, one-way repeated-measures ANOVAs revealed significant session effects (pre-5 min: F (2,62) = 7.22, p = 0.002; warm-up: F (2,62) = 11.57, p = 0.000; cool-down: F (2,62) = 8.99, p = 0.000). Post hoc analyses indicated that FAS values during S30 were significantly higher than those during S1 across all three periods (all p < 0.01, ES = 0.42–0.91), whereas no significant differences were observed between S1 and S15 or between S15 and S30 (all p > 0.05). During the exercise period, FAS was further analyzed using a two-way repeated-measures ANOVA with Session (S1, S15, S30) and Interval (1–6) as within-subject factors. The analysis revealed a significant main effect of Session (F (2,62) = 28.06, p = 0.000), a significant main effect of Interval (F (5,155) = 165.76, p = 0.000), and a significant Session × Interval interaction (F (10,310) = 3.31, p = 0.0004). Post hoc comparisons showed that FAS values were lower in S1 than in S15 and S30 across work intervals 1–6 (all p < 0.01, ES = 0.55–1.08). In addition, differences between S15 and S30 were smaller and limited to later work intervals.

### 3.3 RPE responses

RPE responses during the three Lv-HIIE sessions are shown in Figure 4C. Changes in RPE were examined separately for different periods. During the pre-5 min, warm-up, and cool-down periods, one-way repeated-measures ANOVAs revealed significant session effects (pre-5 min: F (2,62) = 5.20, p = 0.008; warm-up: F (2,62) = 11.28, p = 0.000; cool-down: F (2,62) = 26.15, p = 0.000). Post hoc analyses indicated that RPE values during S1 were significantly higher than those during S30 across all three periods (all p < 0.01, ES = 0.46–0.88), whereas differences between S1 and S15 and between S15 and S30 were smaller and not consistently significant (all p > 0.05). During the exercise period, RPE was further analyzed using a two-way repeated-measures ANOVA with Session (S1, S15, S30) and Interval (1–6) as within-subject factors. The analysis revealed a significant main effect of Session (F (2,62) = 49.35, p = 0.000), a significant main effect of Interval (F (5,155) = 145.65, p = 0.000), and a significant Session × Interval interaction (F (10,310) = 6.34, p = 0.000). Post hoc comparisons revealed that RPE was higher in S1 than in S15 across all work intervals (p < 0.01, ES = 0.62–1.21) and higher than in S30 at work intervals 1–6 (p < 0.001, ES = 0.78–1.34). In addition, RPE in S15 was higher than in S30 at later work intervals (intervals 4–6) (p < 0.01, ES = 0.41–0.76). Across sessions, RPE increased significantly across work intervals (all p < 0.001); however, the onset of this increase occurred progressively later with training (S1: from interval 1; S15: from interval 2; S30: from interval 3). At the final work interval, RPE reached the highest values during S1, followed by S15 and S30.

### 3.4 PRS responses

PRS responses during the three Lv-HIIE sessions are shown in Figure 4D. Changes in PRS were analyzed using a two-way repeated-measures ANOVA with Session (S1, S15, S30) and Interval (rw, r1–r5) as within-subject factors. The analysis revealed a significant main effect of Session (F (2,62) = 65.10, p = 0.000), a significant main effect of Interval (F (5,155) = 96.61, p = 0.000), and a significant Session × Interval interaction (F (10,310) = 11.43, p = 0.000). Post hoc comparisons revealed that PRS values were consistently lower in S1 than in S15 across all intervals (all p < 0.01, ES = 0.82–1.11) and lower than in S30 across all intervals (all p < 0.001, ES = 0.91–1.64). In addition, PRS values in S15 were lower than those in S30 at later intervals (r3–r5) (all p < 0.01, ES = 0.89–1.14). Across sessions, PRS declined significantly across intervals (all p < 0.001); however, the magnitude and timing of this decline differed between sessions, with higher recovery ratings maintained for longer durations during S30 compared with S15 and S1.

### 3.5 Exercise enjoyment responses

**FIGURE 5:**
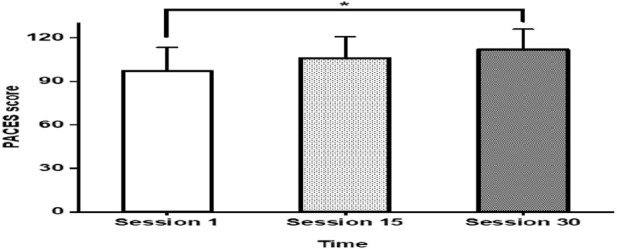
PACES at S1, S15 and S30 Abbreviations: PACES = physical activity enjoyment scale *Significant main effect of time for session (P < 0.001). Error bars are presented as standard deviation.

Changes in post-exercise enjoyment across sessions are shown in Figure 5. The main effect of session was observed (F (1.72, 53.26) = 16.87, p < 0.001). Post-enjoyment scores were significantly lower in S1 compared to S15 and S30 (all p < 0.001; PACES: 97.03 ± 16.59, 105.59 ± 14.82, and 110.84 ± 14.75, respectively; all ES > 0.67). Mean changes (Δ) were 8.56 ± 22.25 between S1 and S15, 13.81 ± 22.20 between S1 and S30, and 5.25 ± 20.91 between S15 and S30.

### 3.6 Within- and between-session comparisons of ACTH and cortisol

Hormonal responses within sessions were examined across the three time points (Pre, Immediately-post, and 30 min-post) during Sessions 1, 15, and 30 (Table 3). For ACTH, significant time-point differences were observed within all sessions, with concentrations increasing from pre-exercise to immediately post-exercise and subsequently decreasing at 30 min post-exercise (all p < 0.001). In addition, ACTH levels at 30 min post-exercise remained significantly higher than pre-exercise levels in Sessions one and 15 (all p < 0.01), whereas this difference was not significant in Session 30 (p > 0.05). For cortisol, significant time-point differences were also observed within all sessions, characterised by an increase from pre-exercise to immediately post-exercise (all p < 0.001) followed by a decrease at 30 min post-exercise (all p < 0.01). No significant differences were detected between pre-exercise and 30 min post-exercise cortisol levels across sessions (all p > 0.05).

**TABLE 3:** Within-session comparisons of ACTH and cortisol across time points.

ACTH measured (pre-immed) (pg/mL)					
Sessions	Pre	Immediately	Δ ACTH	p	ES
S1	34.84 ± 13.67	77.84 ± 51.19	43 ± 52.98*	<0.001	0.91
S15	38.26 ± 14.24	86.83 ± 59.91	48.57 ± 61.58*	<0.001	0.87
S30	45.35 ± 14.29	96.12 ± 59.91	50.77 ± 61.59*	<0.001	0.91

ACTH measured (immed-post) (pg/mL)					
	Immediately	30min-post	Δ ACTH	p	ES
S1	77.84 ± 51.19	46.43 ± 25.89	31.41 ± 57.36*	0.001	0.62
S15	86.83 ± 59.91	49.43 ± 12.56	37.4 ± 61.21*	<0.001	0.68
S30	96.12 ± 59.91	51.12 ± 22.35	45 ± 63.94*	<0.001	0.83

ACTH measured (pre-post) (pg/mL)					
	Pre	30min-post	Δ ACTH	p	ES
S1	34.84 ± 13.67	46.43 ± 25.89	11.59 ± 29.28*	0.009	0.49
S15	38.26 ± 14.24	49.43 ± 12.56	11.17 ± 18.99*	<0.001	0.76
S30	45.35 ± 14.29	51.12 ± 22.35	5.77 ± 26.53	0.14	0.27

Cortisol measured (pre-immed) (ng/mL)					
	Pre	Immediately	Δ cortisol	p	ES
S1	311.98 ± 299.23	429.48 ± 358.87	117.5 ± 467.25*	<0.001	0.74
S15	327.85 ± 306.38	453.37 ± 356.71	125.52 ± 470.22*	<0.001	0.69
S30	353.52 ± 366.91	562.45 ± 471.54	208.93 ± 597.47*	<0.001	0.82

Cortisol measured (immed-post) (ng/mL)					
	Immediately	30 min-post	Δ cortisol	p	ES
S1	429.48 ± 358.87	326.78 ± 282.86	102.7 ± 456.94*	0.003	0.57
S15	453.37 ± 356.71	351.5 ± 316.6	101.87 ± 476.95*	0.009	0.49
S30	562.45 ± 471.54	364.19 ± 290.42	198.26 ± 553.8*	<0.001	0.65

Cortisol measured (pre-post) (ng/mL)					
	Pre	30min-post	Δ cortisol	p	ES
S1	311.98 ± 299.23	326.78 ± 282.86	14.80 ± 411.76	0.49	0.13
S15	327.85 ± 306.38	351.5 ± 316.6	23.65 ± 440.57	0.17	0.25
S30	353.52 ± 366.91	364.19 ± 290.42	10.67 ± 467.94	0.70	0.07

Values are reported as mean ± standard deviation. P value (P) and effect size (ES). * Significant difference between pre-imme, imme-post and pre-post (Bonferroni *post hoc*, P < 0.05).

Hormonal responses were also compared between sessions (S1, S15, and S30) at each time point (Pre, Immediately-post, and 30 min-post) (Table 4). For ACTH, between-session differences were observed at the pre-exercise time point, with higher concentrations in Session 30 compared with Session 15 (p = 0.02) and Session one (p < 0.001), whereas no difference was detected between Session one and Session 15. No between-session differences in ACTH were detected at the immediately post-exercise or 30 min post-exercise time points. For cortisol, between-session comparisons were not significant at any time point (all p > 0.10).

**TABLE 4:** Between-session comparisons of ACTH and cortisol across time points.

ACTH measured (S1-S15) (pg/mL)					
Time points	Session1	Session15	Δ ACTH	p	ES
Pre	34.84 ± 13.67	38.26 ± 14.24	3.42 ± 19.74	0.15	0.26
Immediately	77.84 ± 51.19	86.83 ± 59.91	8.99 ± 78.8	0.27	0.20
30min-post	46.43 ± 25.89	49.43 ± 12.56	3 ± 28.78	0.53	0.11

ACTH measured (S15-S30) (pg/mL)					
	Session15	Session30	Δ ACTH	p	ES
Pre	38.26 ± 14.24	45.35 ± 14.29	7.09 ± 20.17^	0.02	0.43
Immediately	86.83 ± 59.91	96.12 ± 59.91	9.29 ± 84.73	0.51	0.12
30min-post	49.43 ± 12.56	51.12 ± 22.35	1.69 ± 25.64	0.64	0.08

ACTH measured (S1-S30) (pg/mL)					
	Session1	Session30	Δ ACTH	p	ES
Pre	34.84 ± 13.67	45.35 ± 14.29	10.51 ± 19.78^	<0.001	0.77
Immediately	77.84 ± 51.19	96.12 ± 59.91	18.28 ± 78.80	0.21	0.23
30min-post	46.43 ± 25.89	51.12 ± 22.35	4.69 ± 34.20	0.33	0.18

Cortisol measured (S1-S15) (ng/mL)					
	Session1	Session15	Δ cortisol	p	ES
Pre	311.98 ± 299.23	327.85 ± 306.38	15.87 ± 428.26	0.73	0.06
Immediately	429.48 ± 358.87	453.37 ± 356.71	23.89 ± 506	0.60	0.09
30min-post	326.78 ± 282.86	351.5 ± 316.6	24.72 ± 424.55	0.61	0.09

Cortisol measured (S15-S30) (ng/mL)					
	Session15	Session30	Δ cortisol	p	ES
Pre	327.85 ± 306.38	353.52 ± 366.91	25.67 ± 478.01	0.50	0.12
Immediately	453.37 ± 356.71	562.45 ± 471.54	109.08 ± 591.26	0.11	0.30
30min-post	351.5 ± 316.6	364.19 ± 290.42	12.69 ± 429.63	0.68	0.07

Cortisol measured (S1-S30) (ng/mL)					
	Session1	Session30	Δ cortisol	p	ES
Pre	311.98 ± 299.23	353.52 ± 366.91	41.54 ± 473.46	0.48	0.13
Immediately	429.48 ± 358.87	562.45 ± 471.54	132.97 ± 592.57	0.11	0.30
30min-post	326.78 ± 282.86	364.19 ± 290.42	37.41 ± 405.41	0.43	0.14

Values are reported as mean ± standard deviation. P value (P) and effect size (ES). ^Significant difference between s1-s15, s15-s30 and s1-s30 (Bonferroni *post hoc*, P < 0.05).

### 3.7 Correlations between ACTH and perceptual responses

**FIGURE 6:**
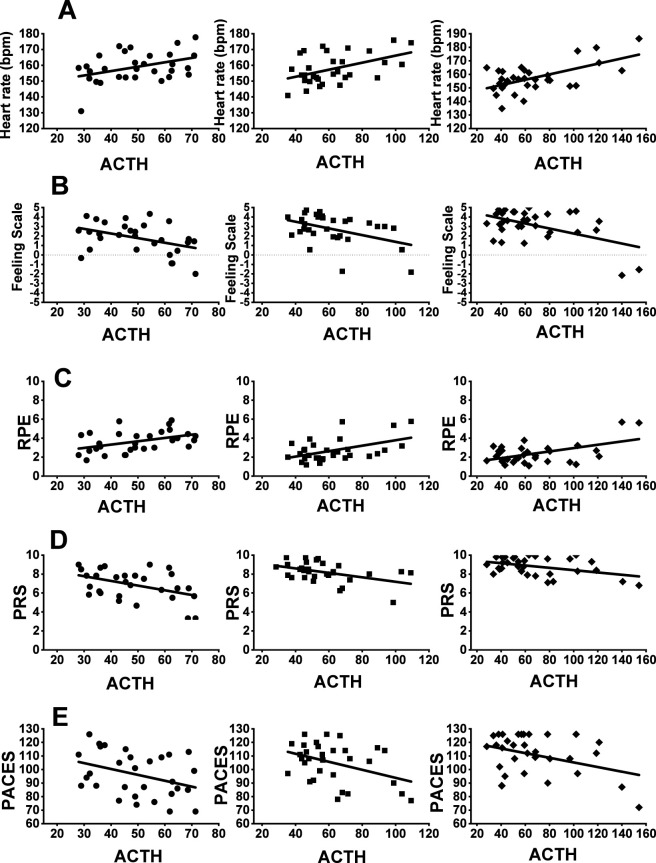
Correlation between ACTH and HR **(A)**, FS **(B)**, RPE **(C)**, PRS **(D)**, PACES **(E)**. Session 1 (s1; ●), Session 15 (s15; ■) and Session 30 (s30; ♦). Abbreviations: Significantly positive correlations **(A) (C)**, Significantly negative correlations **(B) (D) (E)**. ACTH values represent session-averaged concentrations (pre-, immediately post-, and 30-min post-exercise).

Correlations between ACTH and HR during the three sessions of Lv-HIIE protocols are illustrated in Figure 6A. These correlations were calculated using ACTH concentrations averaged across the pre-exercise, immediate post-exercise, and 30-min post-exercise measurements at each session. A moderate positive relationship was observed between ACTH and HR during the work intervals in S1 of Lv-HIIE (p < 0.03, r = 0.40), S15 of Lv-HIIE (p = 0.008, r = 0.47) and S30 of Lv-HIIE (p < 0.001, r = 0.60). Correlations between ACTH and FS during the three sessions of Lv-HIIE protocols are illustrated in Figure 6B. A moderate negative relationship was observed between ACTH and FS during the work intervals in S1 of Lv-HIIE (P < 0.03, r = −0.42), S15 of Lv-HIIE (P < 0.02, r = −0.44) and S30 of Lv-HIIE (P = 0.003, r = −0.51). Correlations between ACTH and RPE during the three sessions of Lv-HIIE protocols are illustrated in Figure 6C. A moderate positive relationship was observed between ACTH and RPE during the work intervals in S1 of Lv-HIIE (P < 0.02, r = 0.43), S15 of Lv-HIIE (P = 0.006, r = 0.48) and S30 of Lv-HIIE (P = 0.002, r = 0.52). Correlations between ACTH and PRS during the three sessions of Lv-HIIE protocols are illustrated in Figure 6D. A moderate negative relationship was observed between ACTH and PRS during the recovery intervals in S1 of Lv-HIIE (P < 0.02, r = −0.44), S15 of Lv-HIIE (P < 0.02, r = −0.45) and S30 of Lv-HIIE (P < 0.02, r = −0.42). Correlations between ACTH and PACES during the three sessions of Lv-HIIE protocols are illustrated in Figure 6E. A weak negative relationship was observed between ACTH and PACES during the post-exercise period in S1 of Lv-HIIE (P < 0.05, r = −0.36) and S30 of Lv-HIIE (all P < 0.04, r = −0.38). A moderate negative relationship was observed during the post-exercise period in S15 of Lv-HIIE (all P < 0.03, r = −0.40).

### 3.8 Correlations between cortisol and perceptual responses

**FIGURE 7:**
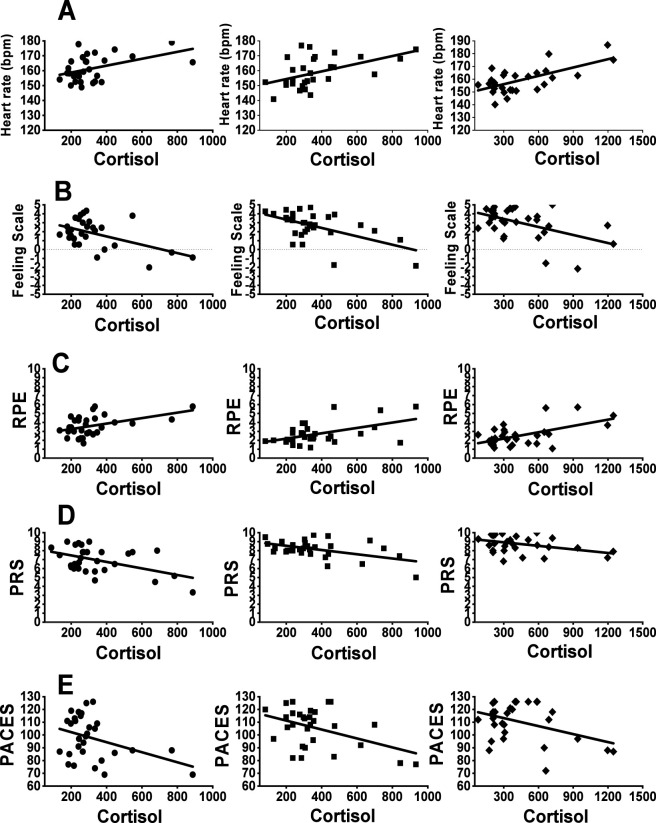
Correlation between cortisol and HR **(A)**, FS **(B)**, RPE **(C)**, PRS **(D)**, PACES **(E)**. Session 1 (s1; ●), Session 15 (s15; ■) and Session 30 (s30; ♦). Abbreviations: Significantly positive correlations **(A) (C)**, Significantly negative correlations **(B) (D) (E)**. Cortisol values represent session-averaged concentrations (pre-, immediately post-, and 30-min post-exercise).

Correlations between cortisol and HR during the three sessions of Lv-HIIE protocols are illustrated in Figure 7A. Correlations were calculated using cortisol concentrations averaged across the pre-exercise, immediate post-exercise, and 30-min post-exercise measurements at sessions 1, 15, and 30. A moderate positive relationship was observed between cortisol and HR during the work intervals in S1 of Lv-HIIE (P < 0.02, r = 0.45) and S15 of Lv-HIIE (P = 0.008, r = 0.47). A strong positive relationship was observed during the work intervals in S30 of Lv-HIIE (P < 0.001, r = 0.61). Correlations between cortisol and FS during the three sessions of Lv-HIIE protocols are illustrated in Figure 7B. A moderate negative relationship was observed between cortisol and FS during the work intervals in S1 of Lv-HIIE (P = 0.004, r = −0.51), S15 of Lv-HIIE (P = 0.001, r = −0.56) and S30 of Lv-HIIE (P = 0.003, r = −0.50). Correlations between cortisol and RPE during the three sessions of Lv-HIIE protocols are illustrated in Figure 7C. A moderate positive relationship was observed between cortisol and RPE during the work intervals in S1 of Lv-HIIE (P < 0.02, r = 0.45), S15 of Lv-HIIE (P = 0.004, r = 0.50) and S30 of Lv-HIIE (P < 0.001, r = 0.58). Correlations between cortisol and PRS during the three sessions of Lv-HIIE protocols are illustrated in Figure 7D. A moderate negative relationship was observed between cortisol and PRS during the recovery intervals in S1 of Lv-HIIE (P = 0.006, r = −0.49), S15 of Lv-HIIE (P = 0.005, r = −0.50) and S30 of Lv-HIIE (P < 0.03, r = −0.40). Correlations between cortisol and PACES during the three sessions of Lv-HIIE protocols are illustrated in Figure 7E. A weak negative relationship was observed between cortisol and PACES during the post-exercise period in S1 of Lv-HIIE (P < 0.04, r = −0.38). A moderate negative relationship was observed during the post-exercise period in S15 of Lv-HIIE (P < 0.02, r = −0.44) and S30 of Lv-HIIE (P < 0.03, r = −0.40).

## 4 Discussion

The key findings of this study are: (1) a 10-week Lv-HIIE intervention significantly improved body composition and cardiorespiratory fitness in adults with overweight and obesity; (2) the intervention was associated with lower heart rate and perceived exertion, higher perceived recovery, more positive affective valence, lower felt arousal, and greater enjoyment across sessions, despite a progressive increase in training volume due to the higher number of work bouts performed per session; and (3) ACTH and cortisol were closely associated with perceptual outcomes, demonstrating positive correlations with heart rate and perceived exertion and negative correlations with affective valence, perceived recovery, and enjoyment.

In the present study, we observed that cardiorespiratory fitness, body fat percentage, and waist-to-hip ratio (WHR) were significantly improved in overweight and obese adults following a 10-week Lv-HIIE intervention (MAS: +0.48 km h^-1^ (+5.0%); *V̇*O_2_max: +0.40 mL kg^-1^·min^-1^ (+1.0%); %BF: −1.11 percentage points (−3.4%); WHR: −0.03 (−3.7%); ES = 0.43–0.57), consistent with beneficial changes observed over a relatively short intervention period. These findings are consistent with the results of a systematic review and meta-analysis, which suggest that HIIE is a time-efficient strategy for improving cardiorespiratory fitness despite low energy expenditure (Wang et al., 2021), and that intervention parameters (such as repetitions, intensity, recovery, and training frequency) are critical for optimizing adaptations. Improvements in body fat percentage and WHR were significant from session one to session 15 (%BF: −0.85 percentage points (−2.6%); WHR: −0.02 (−2.5%); ES = 0.58 and 0.45, respectively) but then plateaued with non-significant changes (%BF: −0.26 percentage points; WHR: −0.01; ES = 0.14 and 0.21), consistent with compensatory patterns described by Gelman et al. (2022). No significant changes in body mass or BMI were observed after 30 sessions (body mass: −0.26 kg; BMI: −0.09 kg m^-2^; ES = 0.01–0.21), which is consistent with evidence that the effects of HIIE on weight-related outcomes are influenced by intervention mode and frequency (Khodadadi et al., 2023). This outcome may reflect that gains in muscle mass offset losses in fat mass, thereby masking overall changes in body mass and BMI. Therefore, longer or more frequent training may be required to achieve measurable improvements.

Throughout the 10-week Lv-HIIE intervention, participants demonstrated progressive improvements in cardiovascular and affective responses. Work and recovery heart rates decreased across sessions, with *post hoc* comparisons indicating lower %HR values in session 30 compared with sessions 1 and 15 across work and recovery intervals, consistent with session-related changes in heart rate responses across the intervention. Similar patterns have been reported in previous HIIE studies (Dun et al., 2019; Milanović et al., 2015; Shiraev and Barclay, 2012). Affective valence improved markedly from the initial to the final session (ΔFS of approximately +1.6 units; ES > 1.19), reflecting more pleasurable feelings over time. In contrast, felt arousal declined progressively, with higher levels in session one and lower levels in session 30 (ΔFAS of approximately −0.9 units; ES = 0.50–1.32), suggesting reduced arousal alongside increased positive affective valence, consistent with the Dual-Mode Theory (Ekkekakis et al., 2005).

Perceived exertion and recovery improved considerably across the intervention period, with RPE significantly reduced (ΔRPE of approximately −1.5 units; ES > 1.58) and perceived recovery significantly improved from session 1 to 30 (ΔPRS of approximately +1.2 units; ES = 0.91–1.64). These findings are consistent with evidence that repeated exposure and familiarity with HIIE reduce perceived exertion and enhance psychological responses (Frazão et al., 2016; Farias-Junior et al., 2019). Post-exercise enjoyment also increased significantly (ΔPACES of approximately +13.8 units; ES > 0.43), consistent with prior HIIE studies (Oliveira et al., 2018; Stork et al., 2017) and theories linking affective responses to enjoyment (Raedeke, 2007). Additional explanations may include a rebound effect following intense effort (Bixby et al., 2001) and the progressive increase in work intervals, which provided greater challenge and enhanced perceived success (Teixeira et al., 2022).

In addition to the session-related adaptations observed during exercise, the present study also demonstrates clear subacute responses to a single Lv-HIIE session, which varied across training sessions. Immediately post-task, participants exhibited elevated heart rate and perceived exertion, whereas partial recovery was evident 30 min post-exercise, as reflected by lower heart rate values and improved perceptual responses. Importantly, these subacute responses were modulated across sessions. Compared with session 1, both the immediate and 30-min post-task responses were attenuated at session 15 and further at session 30, indicating a reduced acute physiological and perceptual strain and a faster post-exercise recovery with training progression. This pattern is consistent with the session-related decreases in work and recovery heart rates, as well as the concurrent reductions in perceived exertion and improvements in perceived recovery observed in the present study. Collectively, these findings suggest that repeated exposure to Lv-HIIE induces chronic adaptations and modifies the acute recovery profile following individual sessions, reflecting an improved tolerance to the exercise stimulus over time. Similar attenuation of acute exercise responses and accelerated recovery with training adaptation have been reported in previous HIIE studies (Buchheit and Laursen, 2013; Milanović et al., 2015; Dupont et al., 2010).

Following the 10-week exercise intervention, significant changes were observed in psychophysiological stress characteristics during the exercise sessions. Specifically, ACTH and cortisol levels increased significantly immediately after exercise and subsequently decreased 30 min post-exercise (Table 3). This time-dependent pattern is consistent with previous reviews demonstrating that high-intensity exercise elicits transient elevations in stress-related hormones followed by post-exercise recovery (Torres et al., 2021).

In this context, ACTH and cortisol were examined in the present study as markers of systemic neuroendocrine load, reflecting activation of the hypothalamic–pituitary–adrenal (HPA) axis in response to overall psychophysiological stress rather than localized muscular fatigue or isolated cardiorespiratory strain. All correlation analyses were performed using mean ACTH and cortisol concentrations averaged across the exercise session for each condition. In this study, we found that ACTH and cortisol levels were moderately positively correlated with heart rate and perceived exertion (Figures 6A,C, 7A,C), indicating that higher stress hormone levels were associated with elevated HR and greater perceived exertion. These findings are consistent with previous evidence linking cortisol to psychosocial stress and emotional stress variables (Gaffey and Wirth, 2014; Wilson et al., 2011; Popp et al., 2015; Campbell and Ehlert, 2012). Tan and Long (2010) similarly observed significant correlations between post-training cortisol and RPE in synchronized swimmers. Furthermore, the correlations of ACTH and cortisol with HR and RPE appeared to strengthen progressively across exercise sessions. This pattern suggests dynamic associations between physiological and perceptual responses and ACTH/cortisol fluctuations across sessions, aligning with Mishica et al. (2021), who reported strengthened HRV–cortisol correlations over 7 weeks, and Rowell et al. (2018), who found that higher training loads were associated with stronger cortisol–RPE relationships.

Conversely, moderate negative correlations were observed between ACTH and cortisol levels and affective valence, perceived recovery, and enjoyment (Figures 6B,D,E, 7B,D,E) indicating that higher hormone levels were associated with reduced positive affect, impaired recovery perception, and lower enjoyment. This finding adds new evidence to the limited literature, as most prior studies have not examined these associations during exercise. Although some studies reported positive associations between cortisol and affective states, as shown in cross-country runners (Rudolph and McAuley, 1998), our results suggest a different trajectory, consistent instead with evidence that prolonged stress diminishes recovery and emotional wellbeing (Rector et al., 2019). Notably, the strongest negative correlations appeared at session 15, possibly reflecting higher cumulative training demands at mid-intervention. Rogers (2022) reported that cumulative exercise stress elevates cortisol and impairs mood, supporting our interpretation. Interestingly, the negative correlations weakened after session 15, with weaker associations between stress hormone levels and affective responses and recovery observed in later sessions.

Several strengths and limitations should be acknowledged in the present study. One strength lies in the sample population, as participants were overweight or obese and physically inactive, thereby increasing the generalizability of the findings to high-risk groups. Another strength is the ecological validity of the intervention, since the Lv-HIIE protocol involved outdoor running that did not require specialized equipment. Furthermore, this study simultaneously assessed physiological outcomes (e.g., *V̇*O_2_max, MAS, %BF, WHR, HR), psychophysiological stress markers (ACTH, cortisol), and perceptual responses (FS, FAS, RPE, PRS, PACES), offering a more comprehensive perspective than many previous studies.

Nevertheless, some limitations should be noted. This study employed a single-arm, non-randomized design without a control group, which precludes causal inference. As such, the observed changes and associations cannot be attributed solely to the Lv-HIIE intervention. In addition, the relatively small sample size and 10-week duration limit the generalizability and long-term implications of the results. The sample consisted primarily of female participants, and sex-specific factors such as menstrual cycle phase and associated hormonal variability were not controlled. This may have influenced ACTH and cortisol responses and should be considered when interpreting the neuroendocrine findings. Furthermore, the large number of correlation analyses conducted across multiple sessions and perceptual outcomes may increase the risk of Type I error. Therefore, the observed associations should be interpreted with caution. In addition, the correlational findings should be considered exploratory in nature, and caution is warranted when interpreting individual correlation coefficients, even when similar patterns are observed across sessions. Moreover, only one type of Lv-HIIE protocol (outdoor running) was examined; other modalities (e.g., cycling, self-paced formats) and variations in work-to-recovery ratios may produce different psychophysiological and perceptual outcomes. Finally, the absence of follow-up assessments prevents conclusions regarding the persistence of the observed adaptations. Therefore, future studies should adopt randomized controlled trial designs, extend intervention duration, and explore different models and population subgroups to refine Lv-HIIE strategies for overweight and obese adults.

## 5 Conclusion

In conclusion, our data extend previous study by showing that a 10-week Lv-HIIE intervention in overweight-to-obese adults was associated with improvements in cardiorespiratory fitness and body composition, including reductions in body fat percentage and waist-to-hip ratio (ES = 0.43–0.57) and increases in *V̇*O_2_max and MAS. Despite progressively increasing training demands, participants reported lower perceived exertion (RPE, ES > 1.58), greater perceived recovery, more positive affective valence (ES > 1.19), lower arousal, and higher enjoyment (ES > 0.43) across the intervention period. ACTH and cortisol levels were dynamically associated with these perceptual responses, showing positive correlations with heart rate and exertion and negative correlations with affective valence, recovery, and enjoyment, with the strength of these associations varying across sessions. Although long-term behavioral outcomes were not assessed, the observed improvements in perceptual tolerance and affective responses during training suggest that Lv-HIIE may be experienced as more tolerable over repeated sessions. Overall, these findings highlight Lv-HIIE as a practical and time-efficient exercise approach associated with both physiological benefits and more positive exercise experiences in adults with excess body weight.

## Data Availability

The raw data supporting the conclusions of this article will be made available by the authors, without undue reservation.
